# Cardioprotective Effects of SIRT6 in a Mouse Model of Transverse Aortic Constriction-Induced Heart Failure

**DOI:** 10.3389/fphys.2017.00394

**Published:** 2017-06-13

**Authors:** Yongming Li, Xianda Meng, Wenguang Wang, Fu Liu, Zhiru Hao, Yang Yang, Jinbo Zhao, Wensi Yin, Lijuan Xu, Ruiping Zhao, Jiang Hu

**Affiliations:** ^1^Department of Cardiology, Baotou Central HospitalBaotou, China; ^2^Department of Cardiology, Dalian (Municipal) Friendship HospitalDalian, China; ^3^Department of Institution of Interventional and Vascular Surgery, Tongji UniversityShanghai, China; ^4^Tanslational Medicine Center, Baotou Central HospitalBaotou, China

**Keywords:** sirtuin, telomerase reverse transcriptase, telomere repeat binding factor-1, cardiovascular diseases

## Abstract

SIRT6, a member of the NAD (+)-dependent class III deacetylase sirtuin family, plays important roles in the maintenance of cardiovascular homeostasis. Telomere shortening is a risk factor for age-associated diseases, including heart disease. In the present study, we investigated the role of SIRT6 and telomerase in a mouse model of transverse aortic constriction (TAC)-induced heart failure. SIRT6, telomerase reverse transcriptase (TERT), and telomere repeat binding factor (TRF)-1 were significantly downregulated in TAC mice compared with their expression in sham-operated mice. Lentiviral vector-mediated overexpression of SIRT6 upregulated TERT and TRF1 and increased the survival of mice after TAC. Echocardiography and hemodynamic measurements as well as histological analyses indicated that SIRT6 overexpression attenuated TAC-induced heart dysfunction and decreased TAC-induced cardiac inflammatory responses, reducing cardiac fibrosis and decreasing infarct size. Taken together, our findings indicate that SIRT6 protects the myocardium against damage and this effect may be mediated by the modulation of telomeres. Our findings linking SIRT6 and telomere integrity in the heart warrant further investigation into the underlying mechanisms and support SIRT6 as a promising therapeutic target for the treatment of cardiovascular diseases.

## Introduction

Sirtuins or silent information regulator (SIR) proteins are NAD-dependent histone deacetylases that function as regulators of cellular homeostasis through their involvement in energy metabolism, genomic stability, inflammation, oxidative stress, and senescence (Kupis et al., 2016). Seven homologs of yeast Sir2 have been identified, and they differ in subcellular localization, enzymatic activity, and targets. SIRT2 which is present predominantly in the cytoplasm, colocalizes with microtubules and is involved in the deacetylation of the main component of microtubules, α-tubulin. SIRT3 is a positive regulator of mitochondrial activity by deacetylating and activating components of the electron transport chain and acetyl-CoA synthetase. SIRT3 is also involved in the defense against oxidative stress. SIRT4, which is located in the mitochondrial matrix, suppresses the secretion of insulin and modulates ATP synthesis. SIRT5 is localized in the mitochondrial matrix, and deacetylates and activates carbamoyl synthetase 1, which catalyzes the first rate-limiting step of the urea cycle. SIRT7 interacts with and downregulates hypoxia-inducible factors HIF-1α and HIF-2α and maintains the malignant transformation of cells through H3K18 deacetylation. SIRT7 also regulates mitochondrial homeostasis through GA binding protein β1, a regulator of mitochondrial biogenesis and function.

SIRT6, which was recently shown to have a more significant association with increased lifespan than other sirtuins, is a nuclear sirtuin localized to heterochromatin that functions in the deacetylation of many critical genes (Hall et al., 2013). SIRT6 represses the activity of transcription factors involved in aging and inflammation, such as NF-κB, c-JUN, and HIF-1α (Zhong and Mostoslavsky, 2010). SIRT6 is highly expressed in endothelial cells, where it confers protection against telomere and genomic DNA damage, thereby preventing the onset of premature senescence (Cardus et al., 2013). SIRT6 prevents cardiac hypertrophy and heart failure, and it contributes to the maintenance of endothelial homeostasis, thus delaying vascular aging (Sundaresan et al., 2012; Kida and Goligorsky, 2016).

Vascular aging involves senescence of endothelial and vascular smooth muscle cells, which can be caused by telomere attrition. Telomeres are chromatin structures that protect the ends of chromosomes from recombination and degradation, thereby preserving DNA integrity and stability (Blackburn, 2001; Blasco, 2005). Telomeric DNA consists of noncoding double stranded repeats of a DNA sequence (TTAGGG in vertebrates), the synthesis of which is catalyzed by telomerase. Telomerase consists of a catalytic subunit (telomerase reverse transcriptase or TERT) and the telomerase RNA component (TERC), which serves as a template for the synthesis of telomeric DNA. Telomeric length and structure are controlled by the binding of telomeric repeat binding factors 1 and 2 (TRF1 and TRF2) to the TTAGGG repeat and the formation of protein complexes. Telomere shortening has been linked to endothelial cell senescence, atherosclerosis, end-stage heart failure, and cardiac hypertrophy among other cardiovascular associated disorders, although whether telomere shortening is a cause or consequence of cardiovascular disease remains unclear (Fuster and Andres, 2006).

In the present study, we investigated the role of SIRT6 and TERT in cardiovascular disease in a mouse model of transverse aortic constriction (TAC)-induced heart failure and explored the underlying mechanisms.

## Materials and methods

### Transverse aortic constriction (TAC)

All animal experiments were approved by the medical ethics committee of Central Hospital of Baotou City, and performed in conformance with the Principles of Laboratory Animal Care (National Society for Medical Research) and in accordance with National Institutes of Health guidelines. Mice used in the study were C57BL/6 mice (8–10 weeks of age) purchased from Shanghai Laboratory Animal Center (Shanghai, China) and housed in a facility with a 12-h light/dark cycle at a controlled temperature and humidity with free access to food and water.

The TAC model was generated by tying a suture around the transverse aorta over a 27-gauge blunted needle to cause occlusion of the aorta. After withdrawing the needle, a stenotic aortic lumen was generated. Mice were euthanized by removing the heart under anesthesia. Sham-operated mice without TAC were used as controls.

### Lentiviral construct and production

For SIRT6 overexpression, lentiviral vectors were constructed by amplifying the cDNA of SIRT6 by PCR using specific primers (forward primer: 5′-CCGCTCGAGGAAGCGGCCTCAACAAGG-3′, and reverse primer: 5′-CGCGGATCCGTGGTTCCTTCAAGTTCCCC-3′) and subcloning into the pLVX-IRES-puro lentiviral vector using Xho I and BamH I restriction sites (underlined). High titer lentiviruses were generated by transfection of human embryonic kidney 293 cells with recombined vectors. Approximately 48 h post-transfection, cells were harvested, purified by centrifugation, and stored at −80°C for study.

Mice were injected intravenously via the tail vein with 100 μl pLVX-IRES-puro vector (vector) or vector expressing SIRT6 (pSIRT6) lentivirus plasmids (lentivirus at 100 million virus/100 μl PBS/mouse) at 2 weeks and 24 h before TAC or sham surgery. Myocardial tissue samples were collected at 28 days after surgery. Mice without lentivirus injection served as the control (Ctrl).

### Echocardiographic and hemodynamic measurements

Echocardiographic measurements were obtained using a Vevo2100 imaging system (VisualSonics, Toronto, Canada) with a 22–25 MHz linear transducer probe as described previously (Balasubramanian et al., 2015). LV end-diastolic volume (EDV) and end-systolic volume (ESV) were calculated using the Simpson method of disks. Ejection fraction (EF) was determined using the formula EF (%) = (EDV−ESV)/EDV × 100. Left ventricular end-diastolic diameter (LVEDD) and end-systolic diameter (LVESD) were measured at one-dimensional mode according to the American Society of Echocardiography guidelines. LV fractional shortening (FS) was calculated with the following formula: FS (%) = (LVEDD − LVESD)/LVEDD × 100. The maximal rates of rise and fall in LV pressure (+dP/dt, −dP/dt) were recorded as previously described (Toldo et al., 2011). The investigator performing and reading the echocardiogram was blinded to the treatment allocation.

### Quantitative real-time RT-PCR (qRT-PCR)

For quantitative real-time RT-PCR (qRT-PCR), total RNA was isolated from myocardial tissue samples using the TRIzol reagent (Invitrogen, USA) and reverse transcribed using the SuperScript III kit (Invitrogen) according to the manufacturer's instruction. The PCR primers used were as follows: SIRT6 forward, 5′-CCT GGT CAG CCA GAA CGT AG-3′, and reverse, 5′-GTG TCT CTC AGC TCC CCT CT-3′; GAPDH forward, 5′-CCA TCT TCC AGG AGC GAG AC-3′, and reverse, 5′-GCC CTT CCA CAA TGC CAA AG-3′. qRT-PCR reactions were performed using SYBR Green reagents (Invitrogen) and on a MyiQ Single-Color Real-Time PCR Detection System (Bio-Rad Laboratories, USA). PCR conditions were 95°C for 10 min, then 40 amplification cycles of 95°C for 10 s and 72°C for 30 s. Reactions were performed in triplicate, and the level of mRNA was normalized to that of GAPDH using the 2^−ΔΔCt^ method.

### Western blot analysis

For western blot analysis, mouse myocardial tissues from infarcted area of TAC groups and anterior left ventricular of sham operated groups were homogenized in radio-immunoprecipitation assay buffer (Beyotime, Shanghai, China) and protein concentration was determined using the BCA protein assay kit (Beyotime). Aliquots containing 20 μg of protein were separated by 10% sodium dodecyl sulfatepolyacrylamide gel electrophoresis and transferred to nitrocelullose membranes (Pall Corporation, USA). Membranes were incubated in 5% nonfat milk in Tris-buffered saline with Tween 20 (TBST) for 1 h at room temperature, followed by incubation in primary antibodies at 4°C overnight. The primary antibodies used and their dilutions were as follows: anti-SIRT6, 1:2,000; anti-α-tubulin, 1:2,000; anti-TERT, 1:1,000; anti-TRF1, 1:1,000; anti-collagen I, 1:5,000; anti-FN, 1:1,000; anti-α-tubulin, 1:1,000; β-actin, 1:2,000 (all from Abcam, Cambridge, United Kingdom). After washing in TBST, membranes were incubated with the appropriate horseradish peroxidase-conjugated secondary antibodies at room temperature for 1 h. Signal detection was performed using the SuperSignal ECL system (ThermoScientific, Waltham, Massachusetts) and bands were analyzed by ImageJ software. Band intensity was normalized to that of α-tubulin or β-actin.

### Assessment of infarct size and survival

Infarct size was determined using the 2,3,5-triphenyltetrazolium chloride (TTC) staining. Briefly, mice were euthanized 28 days after surgery. The hearts were rapidly removed, frozen, and sliced into transverse sections (~2 mm). The heart slices were stained with TTC, fixed, and photographed using a digital camera. The infarct area (pale white) in the LV was determined using Meta Morph software (version 6.0, Universal Imaging Corporation). Infarct size was presented as percentage of total LV surface. Survival analysis was performed by daily cage inspection for up to 28 days after surgery.

### Histology and immunohistochemistry

For histological analyses, mice were euthanized and the hearts were cut into transverse segments, fixed in 10% formaldehyde, and embedded in paraffin. Sections were cut at a thickness of 10 μm and either stained with Masson's trichrome to measure fibrotic areas using ImagePro software. The following antibodies were used for immunohistochemistry analyses: anti-Ly-6G (1:100, Abcam) to detect neutrophils and anti-CD68 (1:200, Abcam) to detect macrophages. Quantitative assessment of neutrophil and macrophage density was performed by counting the number of Ly6G- and CD68-immunoreactive cells, respectively, in a double-blind fashion from five different fields of the infarcted area at 400 × magnification. For immunofluorescent staining, tissue sections were incubated with anti-SIRT6 antibody (1:100, Abcam) overnight and secondary antibody for 90 min. DAPI solution was then added for 2 min to stain the nuclei. Images were captured using a Laser-Scanning Confocal Microscope (Olympus FluoView™ FV1000, Tokyo, Japan).

### Enzyme-linked immunosorbent assay (ELISA) analysis

TNF-α, IL-1β, and IL-10 concentrations in mouse hearts were assessed by ELISA using a commercial kit (R&D System, Minneapolis, MN, USA), according to manufacturer's instruction. Tissues from infarcted area of TAC groups or anterior left ventricular of sham operated group were homogenized in ice-cold PBS buffer containing protease inhibitor cocktail (21 mmol/L leupeptin and 3.1 nmol/L aprotinin), and total proteins were extracted using a protein extraction kit (Pierce, Rockford, IL, USA). Results are expressed as pg/mg tissue.

### Statistical analysis

Data are presented as the mean ± standard deviation. Data were analyzed with SPSS (version 22.0; SPSS, Chicago, USA) or GraphPad Prism (version 5.0; GraphPad, La Jolla, USA). Student's *t*-test was applied to determine statistically significant differences between groups. One-way analysis of variance followed by Tukey's *post-hoc* test was applied to determine the significance among groups. *P* < 0.05 was considered statistically significant.

## Results

### SIRT6 and telomerase associated proteins are downregulated in the myocardium of TAC mice

**Figure 1:**
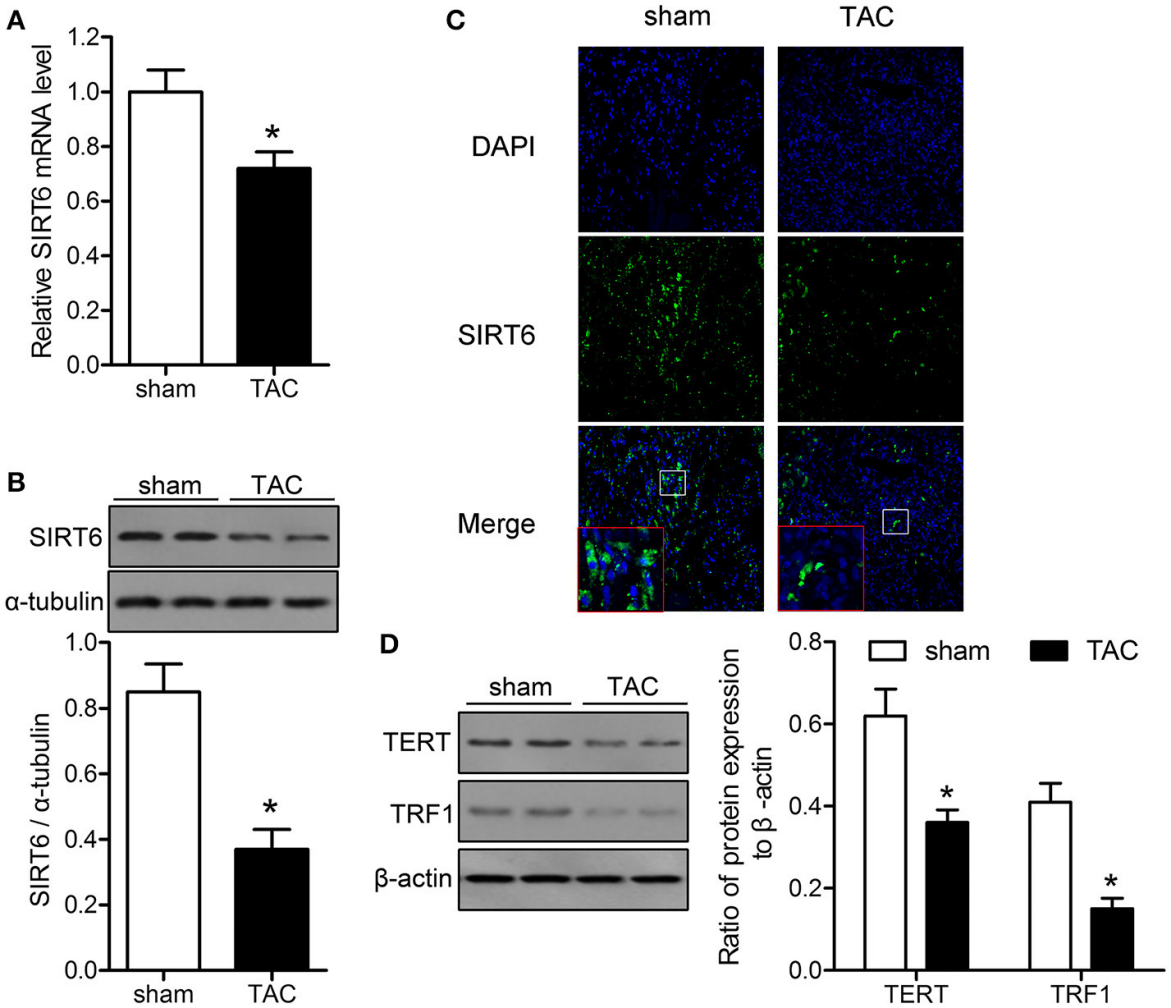
SIRT6 and telomerase associated proteins are downregulated in the myocardium of transverse aortic constriction (TAC) mice. TAC or sham surgery was performed in mice, and myocardial tissue samples were collected at 28 days after surgery. **(A)** SIRT6 mRNA expression in myocardial tissues was determined by qRT-PCR and normalized to GAPDH expression. ^*^P < 0.05 vs. sham group. **(B)** Western blot assessment of the expression levels of SIRT6 normalized to α-tubulin expression level. ^*^P < 0.05 vs. sham group. **(C)** Immunofluorescence labeling of SIRT6 (green) in myocardial tissues, nuclei were stained with DAPI (blue). 200 × magnification. **(D)** Western blot assessment of the expression levels of telomere reverse transcriptase (TERT), and telomere repeat binding factor (TRF)1 normalized to β-actin expression levels. ^*^P < 0.05 vs. sham group. *n* = 5 in the sham group, and *n* = 8 in the TAC group.

The levels of SIRT6, TERT, and TRF1 were measured in myocardial tissues of mice exposed to TAC or sham surgery to explore the association of sirtuins and telomere function with TAC-induced heart failure. The results of qRT-PCR and western blotting showed that SIRT6 was significantly downregulated in TAC mice at the mRNA and protein levels compared with the levels in sham-operated mice (Figures 1A,B). Immunofluorescence analysis of myocardial tissues confirmed that SIRT6 was downregulated in TAC compared with sham-operated mice (Figure 1C). Western blot analysis showed that TAC significantly downregulated TERT and TRF1 proteins in myocardial tissues compared with their expression in sham-operated mice (Figure 1D).

### SIRT6 overexpression improves the survival of TAC mice and upregulates tert and TRF1

**Figure 2:**
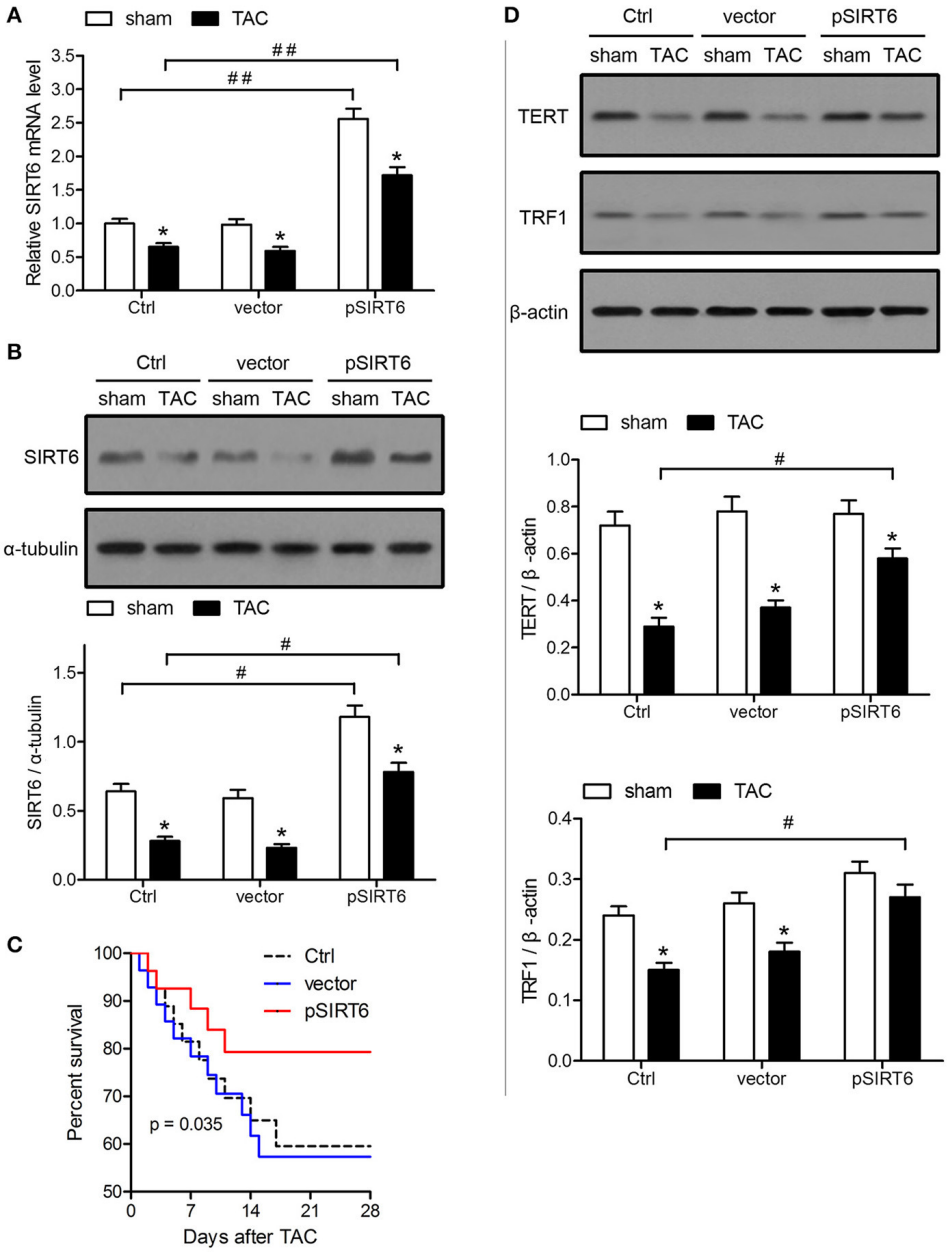
SIRT6 overexpression improves the survival of TAC mice and upregulates TERT and TRF1. Mice were injected intravenously via the tail vein with 100 μl pLVX-IRES-puro vector (vector) or vector expressing SIRT6 (pSIRT6) lentivirus plasmids at 2 weeks and 24 h before TAC or sham surgery. Myocardial tissue samples were collected at 28 days after surgery. Mice without lentivirus injection served as the control (Ctrl). **(A)** SIRT6 mRNA expression in myocardial tissues was determined by qRT-PCR and normalized to GAPDH levels. ^*^P < 0.05 vs. sham group, ^##^*P* < 0.01. *n* = 8–10 per group. **(B)** Protein expression levels of SIRT6 were analyzed by western blotting and normalized to α-tubulin expression levels. ^*^P < 0.05 vs. sham group, ^#^*P* < 0.05. *n* = 8–10 per group. **(C)** Survival analysis of mice treated as indicated each day after TAC. *n* = 30 per group. **(D)** Western blot assessment of TERT and TRF1 expression normalized to β-actin levels. ^*^P < 0.05 vs. sham group, ^#^*P* < 0.05. *n* = 8 per group.

The effect of SIRT6 was further analyzed by injection of a vector expressing SIRT6 lentivirus plasmids into mice before TAC, which resulted in the effective overexpression of SIRT6 mRNA and protein in TAC and sham-operated mice, as indicated by qRT-PCR and western blot analysis (Figures 2A,B). Kaplan-Meier survival analysis showed that mice overexpressing SIRT6 had significantly better survival rates after TAC than control or empty vector-injected mice (Figure 2C). The improved survival occurred in parallel with the upregulation of TERT and TRF1 proteins in TAC mice overexpressing SIRT6 compared with the respective controls (Figure 2D). These results suggested that SIRT6 overexpression protects mice against TAC-induced heart failure by modulating the expression of telomere regulatory proteins.

### SIRT6 overexpression protects against TAC-induced impairment of cardiovascular function

**Figure 3:**
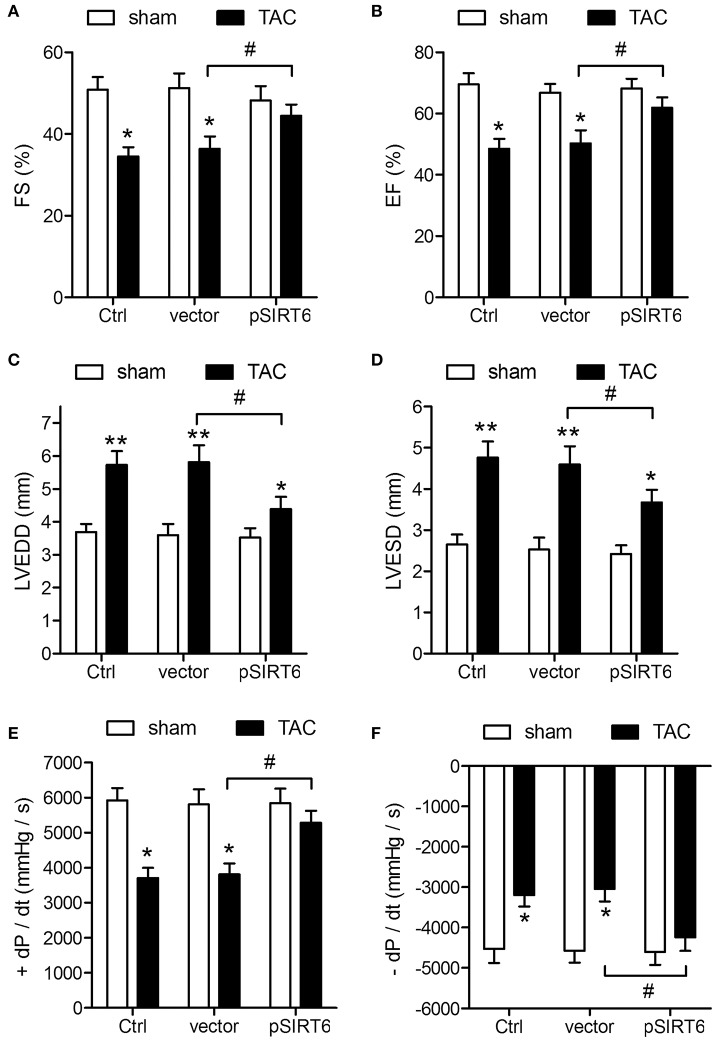
SIRT6 overexpression protects against TAC-induced impairment of cardiovascular function. Echocardiographic and hemodynamic measurements of fractional shortening (FS) **(A)**, ejection fraction (EF) **(B)**, left ventricular end-diastolic dimension (LVEDD) **(C)**, left ventricular end-systolic dimension (LVESD) **(D)**, rates of maximal rise in left ventricular pressure (+dP/dt) **(E)**, and rate of maximal fall in left ventricular pressure (−dP/dt) **(F)** in control, vector and pSIRT6-injected mice at 28 days after TAC or sham surgery. ^*^P < 0.05, ^**^P < 0.01 vs. sham group, ^#^*P* < 0.05. *n* = 12 per group.

The protective effect of SIRT6 was further analyzed by measuring cardiovascular function after TAC. The results of echocardiographic and hemodynamic measurements showed that SIRT6 overexpression attenuated the effects of TAC on decreasing FS and EF and increasing LVEDD and LVESD (Figures 3A–D). SIRT6 overexpression also attenuated the effects of TAC on the maximal fall and rise of left ventricular pressure compared with that in vector mice (Figures 3E,F). Taken together, these results indicated that SIRT6 exerts a protective effect against TAC-induced cardiovascular function impairment.

### SIRT6 overexpression decreases TAC-induced myocardial inflammation

**Figure 4:**
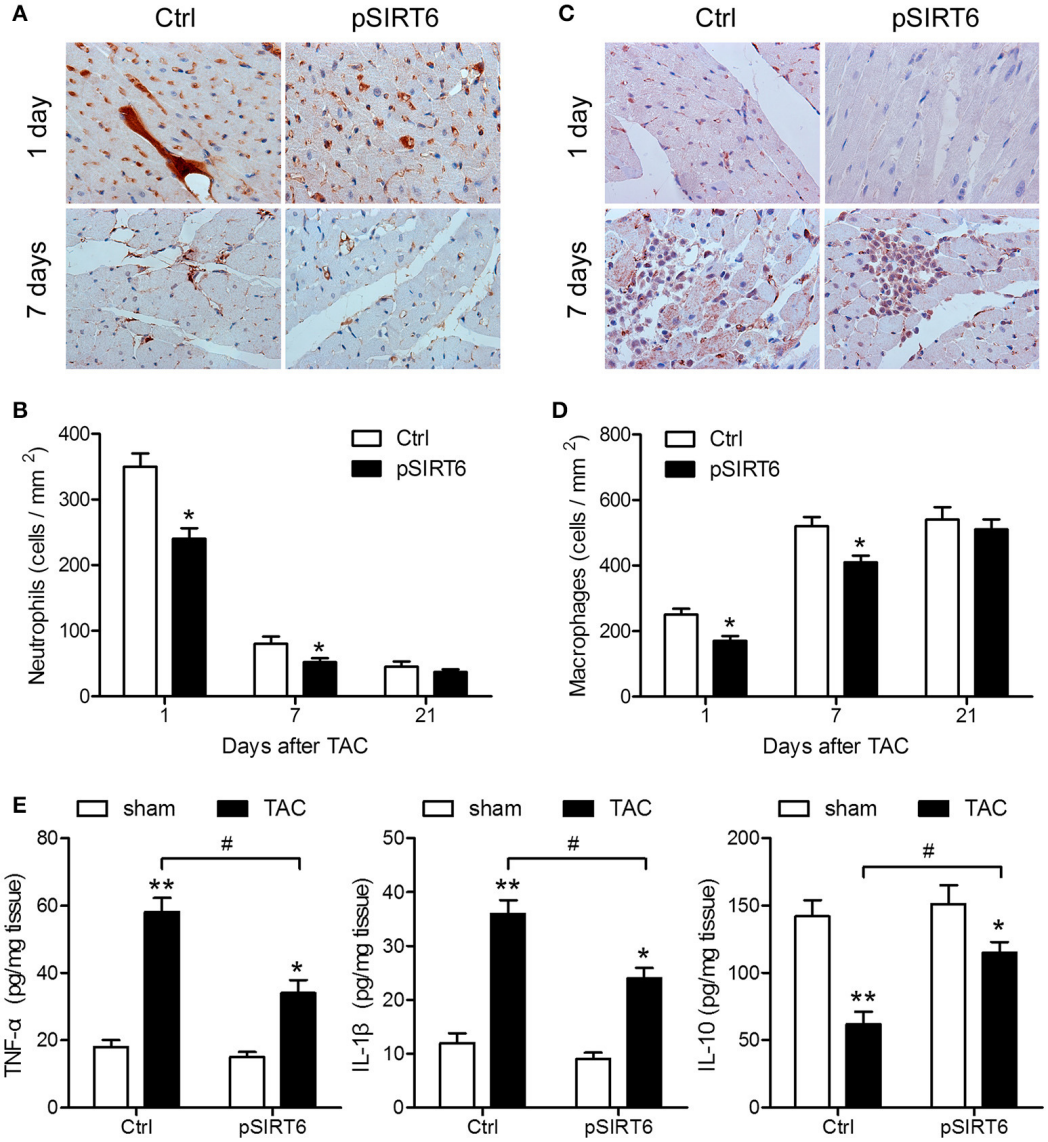
SIRT6 overexpression decreases TAC-induced myocardial inflammation. **(A)** Representative images of neutrophil (Ly6G + cell) infiltration in control and pSIRT6-injected mice at 1 and 7 days after TAC surgery are shown at 400 × magnification. **(B)** Quantification of infiltrated neutrophils per area in frozen sections of infarcted hearts at different time points after TAC surgery. **(C)** Representative images of macrophage (CD68 + cell) infiltration in control and pSIRT6-injected mice at 1 and 7 days after TAC surgery are shown at 400 × magnification. **(D)** Quantification of infiltrated macrophages per area in frozen sections of infarcted hearts at different time points after TAC surgery. ^*^P < 0.05 vs. Ctrl group. *n* = 8 per group. **(E)** Levels of TNF-α, IL-1β, and IL-10 measured in the heart homogenate of control and pSIRT6-injected mice after TAC or sham surgery for 24 h. ^*^P < 0.05, ^**^P < 0.01 vs. sham group, ^#^*P* < 0.05. *n* = 8 per group.

Cardiac inflammation was assessed by measuring neutrophil and macrophage infiltration, which showed that SIRT6 overexpression significantly decreased neutrophil infiltration at 1 day after TAC and attenuated the increase in macrophages at 1 and 7 days after TAC, indicating that SIRT6 decreased cardiac inflammatory responses induced by aortic constriction (Figures 4A–D). In addition, assessment of the levels of TNF-α, IL-1β, and IL-10 by ELISA in the heart homogenate of control and pSIRT6-injected mice after TAC or sham surgery showed that SIRT6 significantly attenuated the TAC-induced upregulation of TNF-α and IL-1β and the downregulation of IL-10 (Figure 4E), suggesting that the effect of SIRT6 was mediated by the modulation of inflammatory factors.

### SIRT6 overexpression reduces infarct size and cardiac fibrosis after TAC

**Figure 5:**
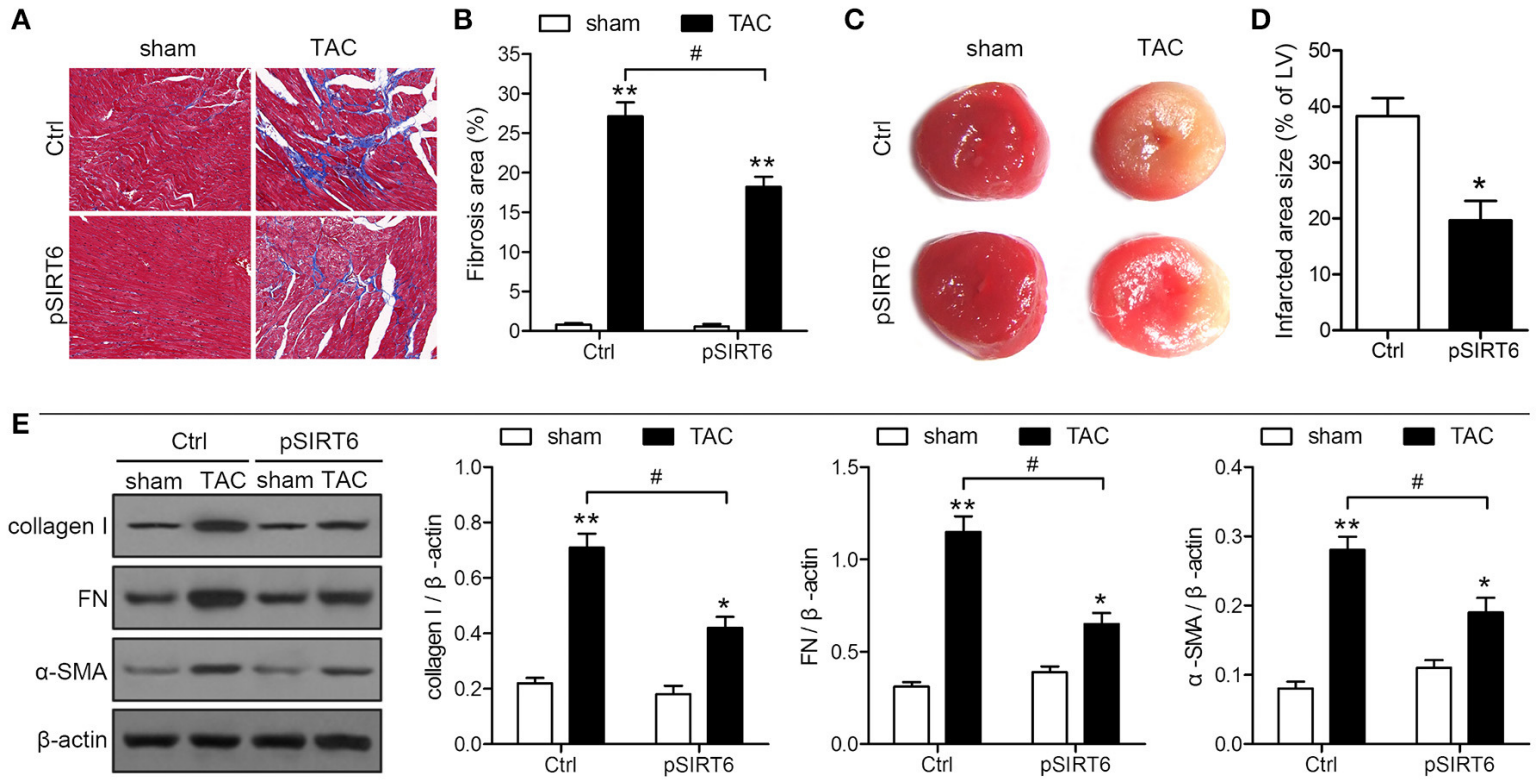
SIRT6 overexpression reduces infarct size and cardiac fibrosis after TAC. Myocardial tissues were collected at 28 days after surgery in control and pSIRT6-injected mice. **(A)** Representative images of left ventricular sections stained with Masson's trichrome at 200 × magnification. Fibrotic areas are stained in blue. **(B)** Quantification of fibrotic areas. ^**^P < 0.01 vs. sham group, ^#^*P* < 0.05, *n* = 8 per group. **(C)** Representative images of middle heart sections stained with TTC. The infarct area stained pale white. **(D)** Quantitative analysis of infarct size in TAC mice from TTC stained images. ^*^P < 0.05 vs. Ctrl group, *n* = 6 per group. **(E)** Protein expression levels of collagen I, fibronectin (FN) and α-SMA were analyzed by western blotting and normalized to β-actin expression levels. ^*^P < 0.05, ^**^P < 0.01 vs. sham group. ^#^*P* < 0.05, *n* = 10 per group.

Histological analysis of sections of the heart by Masson's trichrome staining showed that SIRT6 overexpression significantly attenuated the TAC-induced increase in the areas of fibrosis in mice exposed to TAC compared with sham-operated mice (Figures 5A,B). In addition, heart sections were stained with TTC to determine myocardial infarct size. The results showed that SIRT6 significantly reduced the size of the TAC-induced infarcted area (Figures 5C,D). Western blot analysis showed that SIRT6 significantly attenuated the TAC-induced upregulation of collagen I, fibronectin, and α-SMA in myocardial tissues (Figure 5E). Taken together, these results indicated that SIRT6 protects the myocardium against TAC-induced heart failure, improving heart function, reducing infarct size and cardiac fibrosis, and attenuating inflammatory responses, and these effects may be mediated by the modulation of telomere size and structure.

## Discussion

Heart disease is the leading cause of death worldwide, with approximately 17.5 million deaths from cardiovascular disease reported in 2012 according to World Health Organization estimates (UnitedHealth Group/National Heart, Lung, and Blood Institute Centres of Excellence, 2014). Extensive research into the molecular mechanisms of cardiovascular disease identified a number of risk factors, and studies suggest that heart failure can be prevented or reverted (Sundaresan et al., 2012). Caloric restriction has been identified as a protective factor with the potential toprevent aging-related cardiovascular diseases (Baur et al., 2012). Sirtuins are mediators of the beneficial effects of caloric restriction, and several sirtuins including SIRT1, SIRT3, and SIRT6 are activated in response to caloric restriction (Cohen et al., 2004; Kanfi et al., 2008; Hirschey et al., 2010). In the present study, we examined the role of SIRT6 in TAC-induced heart failure and the involvement telomere integrity in the protective effects of SIRT6 on cardiovascular function.

The results of the present study showed that SIRT6 was downregulated in mice exposed to TAC compared with its expression in sham-operated mice, and ectopic expression of SIRT6 was associated with increased survival in mice undergoing TAC. Functional and morphological studies showed that SIRT6 overexpression attenuated TAC-induced heart damage and decreased inflammation in the hearts of mice exposed to TAC. Several sirtuins have been shown to exert cardioprotective effects. SIRT1 activates endothelial nitric oxide synthase and protects vascular smooth muscle cells against DNA damage, medial degeneration, and atherosclerosis (Nisoli et al., 2005; Gorenne et al., 2013). SIRT3, a mitochondrial sirtuin, promotes antioxidant defense responses and preserves mitochondrial function, thereby protecting against cardiovascular diseases associated with mitochondrial dysfunction such as dilated cardiomyopathy, heart failure, pulmonary hypertension, and endothelial dysfunction (Winnik et al., 2015). SIRT6, which is highly expressed in the heart, plays a protective role in several cardiovascular-related diseases, including cardiac hypertrophy, heart failure, atherosclerosis, myocardial hypoxic damage, and metabolism (Cai et al., 2012; Maksin-Matveev et al., 2015; Liu et al., 2016). SIRT6 is a negative regulator of the myocardial IGF-Akt signaling pathway, the constitutive activation of which leads to cardiac hypertrophy (Sundaresan et al., 2012). Furthermore, SIRT6 is downregulated in myocardial samples from heart failure patients and in mouse models of cardiac hypertrophy, and SIRT6 overexpression protects the heart from the effects of hypertrophic stimuli, which is consistent with our present findings. SIRT6 acts as a transcriptional repressor of NF-κB, thereby protecting cells against vascular inflammation, a key process in atherogenesis (Libby et al., 2009). SIRT6 associates with telomeres and modulates telomeric chromatin. SIRT6 depletion results in abnormal telomere structure, and studies have shown that the histone deacetylase activity of SIRT6 ensures proper chromatin function, including telomere and genome stabilization, gene expression, and DNA repair, thus preventing cellular senescence (Michishita et al., 2008; Beauharnois et al., 2013).

Telomere length plays an important role in aging related diseases such as atherosclerosis and cardiovascular disease. Telomeres are markers of cellular senescence, and myocardial or vascular dysfunction occurring during aging is related to cellular senescence and loss of function (Matthews et al., 2006; Collerton et al., 2007). Telomeres are involved in the development and progression of cardiovascular diseases even independently of aging, as shown by the presence of shortened telomeres in patients with coronary artery disease or myocardial infarction (Brouilette et al., 2003, 2007). Telomere length in vascular cells affects the development of coronary artery disease by mediating smooth muscle cell and endothelial cell senescence (Ogami et al., 2004; Matthews et al., 2006). Telomere length has also been associated with heart failure, and telomere length is inversely proportional to the severity of heart failure (van der Harst et al., 2007). These studies link the integrity of telomeres to the onset and progression of cardiovascular diseases, although some of the underlying mechanisms remain unclear. In the present study, the downregulation of SIRT6 in TAC mice occurred in parallel with the downregulation of TERT and TRF1, and the increased survival in mice overexpressing SIRT6 was accompanied by the upregulation of these telomere-associated proteins, suggesting that the protective effects of SIRT6 are mediated by the regulation of telomere integrity. However, the exact mechanisms mediating the effect of SIRT6 on cardiac function remain unclear. For example, SIRT6 could affect the structure and function of the heart at the cellular level. Crocini et al. recently described the involvement of T-tubule dysfunction on alterations in Ca^2+^ release associated with heart failure (Crocini et al., 2014). SIRT1 was suggested to be involved in the modulation of the sarcoplasmic calcium ATPase SERCA2a, which plays an essential role in the maintenance of Ca^2+^ balance during the cardiac cycle by regulating its transport to the sarcoplasmic reticulum during relaxation (Sulaiman et al., 2010). SERCA2a mRNA is downregulated in patients with dilated or ischemic cardiomyopathy and in aging rat hearts, and downregulation or impaired function of SERCA2a is associated with cardiomyopathy (Tanno et al., 2012). Similarly, SIRT6 could be involved in the regulation of intracellular Ca^2+^ levels in cardiomyocytes and thus in the maintenance of action potentials in the myocardium. Future studies should focus on exploring the mechanisms by which SIRT6 protects the heart in our *in vivo* model of TAC-induced heart failure.

In conclusion, we used a mouse model of TAC-induced heart failure to explore the role of SIRT6 and the modulation of telomere length and structure in the protection against cardiovascular dysfunction. We showed that SIRT6 overexpression increased survival, attenuated TAC-induced heart damage and dysfunction, decreased myocardial inflammation induced by TAC, and reduced the areas of fibrosis and infarct size in TAC mice. These effects were accompanied by the upregulation of TERT and TRF1 and the modulation of inflammatory factors, suggesting that SIRT6 protects against heart failure via a mechanism involving the regulation of telomere integrity and inflammatory responses. The ability of sirtuins to improve responses to aging associated stresses makes them potential therapeutic targets, and sirtuin activating compounds have shown promising results in the treatment of inflammatory and metabolic disorders (Bonkowski and Sinclair, 2016). Therefore, our findings are of particular importance and merit further investigation into the regulatory role of SIRT6 in the myocardium and the pathways involved.

## Author contributions

YL, XM, and RZ designed the research, analyzed the data and drafted the manuscript. YL, WW, and FL performed experiments. ZH, YY, and XM helped for data acquisition and discussion of the data. WY and LX analyzed the data and prepared the figures. JH supervised the whole project. All authors contributed to manuscript writing and edition.

### Conflict of interest statement

The authors declare that the research was conducted in the absence of any commercial or financial relationships that could be construed as a potential conflict of interest.
